# The Role of Open Lung Biopsy in Critically Ill Patients with Hypoxic Respiratory Failure: A Retrospective Cohort Study

**DOI:** 10.1155/2016/8715024

**Published:** 2016-05-04

**Authors:** Abdullah Almotairi, Sharmistha Biswas, Jason Shahin

**Affiliations:** ^1^McGill University, 845 Sherbrooke Street West, Montreal, QC, Canada H3A 2T5; ^2^Department of Pulmonary & Critical Care Medicine, King Fahad Medical City, P. O. Box 59046, Riyadh 11525, Saudi Arabia; ^3^Department of Critical Care and Department of Medicine, Respiratory Division, Respiratory Epidemiology Clinical Research Unit, McGill University Health Centre, 687 Pine Avenue West, Montreal, QC, Canada H3A 1A1

## Abstract

*Background*. The aim of this study was to assess the utility of open lung biopsy in patients with hypoxic respiratory failure of unknown etiology admitted to an ICU and to examine the use of steroid therapy in this patient population.* Methods*. A retrospective cohort study was performed of all consecutive patients admitted to three tertiary care, university-affiliated, ICUs during the period from January 2000 to January 2012 with the principal diagnosis of hypoxic respiratory failure and who underwent an open lung biopsy.* Results*. Open lung biopsy resulted in a diagnostic yield of 68% and in a 67% change of management in patients. A multivariable analysis of clinical variables associated with acute hospital mortality demonstrated that postbiopsy systemic steroid therapy (OR 0.24, 95% C.I 0.06–0.96) was significantly associated with improved survival. Complications arising from the biopsy occurred in 30% of patients.* Conclusion*. Open lung biopsy had significant diagnostic yield and led to major changes in management and aided in end-of-life decision-making in the ICU. Systemic steroid therapy was associated with improved survival. The risk-benefit ratio of open lung biopsy is still unclear, especially given the availability of newer diagnostic tests and possible empirical therapy with steroids.

## 1. Background

Hypoxic respiratory failure is a frequent cause of admission to an intensive care unit (ICU) and contributes significantly to patient morbidity and mortality. Therapeutic interventions consist mainly of supportive care and therapies targeting the underlying etiology of the respiratory failure. Systemic corticosteroids are often administered empirically in selected cases of hypoxic respiratory failure despite the lack of mortality benefit demonstrated in a randomized clinical trial of patients with acute respiratory distress syndrome (ARDS). The utility of performing an open lung biopsy in patients with hypoxic respiratory failure of unknown etiology is still debated as the risk-benefit ratio is unknown [1]. Furthermore, the benefits of steroid therapy in this patient population are also unclear.

The aim of this study was to assess the utility of open lung biopsy in patients with hypoxic respiratory failure of unknown etiology admitted to an ICU. Specifically, we sought to assess whether open lung biopsy yields diagnostic information that influences patient management, the occurrence of adverse outcomes following the procedure, and the benefit associated with steroid administration.

## 2. Methods

### 2.1. Study Design

A retrospective cohort study of all consecutive patients admitted to three tertiary care, university-affiliated, ICUs during the period of January 2000 to January 2012 with the principal diagnosis of hypoxic respiratory failure was performed. Data were abstracted by a trained data collector using a standardized data collection tool. The study was approved by the McGill University Health Centre Research Ethics Board. Due to the retrospective study design, informed consent was waived by the research ethics board.

The patients were identified via each center's ICU database by searching for patients who received surgical open lung biopsies. All patients who met the inclusion criteria were included. In order to assure full patient capture, a second list was generated by the medical archivist in each hospital using the open lung biopsy code and cross-linked with the ICU database lists.

### 2.2. Patient Selection

All patients aged 18 and older who were admitted to the intensive care with acute hypoxic failure and bilateral chest infiltrate and who underwent open lung biopsy were included in the study. Intubation was not a mandatory criterion.

### 2.3. Data Collection

Data were collected on age, sex, severity of illness, PaO_2_/FiO_2_, vasopressor use, mechanical ventilation status, comorbidities, therapies received prior to and after open lung biopsy, diagnostic procedures, ventilator settings, radiographic imaging, pathological results, and complications.

Severity of illness was defined using the APACHE II score [2]. Vasopressor therapy was included if norepinephrine >5 mcg/min for at least one hour or any other vasopressor or inotrope was given at any dose for any amount of time. Mechanical ventilation on the first day of ICU admission was documented. Mechanical ventilation was categorized as either invasive or noninvasive. In addition, mechanical ventilation and oxygenation parameters before and after the biopsy were recorded from respiratory technician notes. This included positive end expiratory pressure levels, PaO_2_/FiO_2_, oxygen saturation, peak inspiratory pressure, respiratory rate, and tidal volumes. Comorbidities were extracted from the patient's medical records.

Data were collected on therapies received, while in the ICU, before and after open lung biopsy. Specifically, data on vasopressors, inotropes, antibiotics, immunosuppressants, and corticosteroids were collected. Steroid use was considered for any dose equivalent to or greater than 10 mg of prednisone daily for one month or more.

All diagnostic procedures before the biopsy relating to diagnostic workup for the hypoxic respiratory failure were collected. This included sputum cultures, blood cultures, bronchoscopic specimens, serum antibody or antigen, or polymerase chain reaction assays. The bronchoscopy reports were noted.

Complications related to surgery were collected from physician and surgeon notes. Air leak was defined as persistent air bubbles in the draining system up to one week after biopsy. Postbiopsy bleeding was defined as any bleeding related to the open lung biopsy that required transfusion of more than two units of packed red blood cells. Impact of open lung biopsy results on the management of patients regarding change of therapy was documented. Change of therapy was defined as the initiation, cessation, or change of dose of an antibiotic, steroid, or immunosuppressive medication. Patients who had withdrawal of life support therapy as a result of information gained from the open lung biopsy were also considered to have had a change of therapy. Timing of biopsy was defined as late if performed 5 days or more after admission to ICU. Of note, data on type of surgical approach utilized for open lung biopsy was not formally collected but the majority of the surgeons tended to prefer an open thoracotomy approach rather than video assisted.

Outcomes that were assessed consisted of diagnostic yield, acute hospital mortality, mean number of ventilated days, ICU length of stay, biopsy related complications, and change of therapy. Diagnostic yield was defined as a result on pathology that led to a specific etiology causing the respiratory failure other than diffuse alveolar damage. Acute hospital mortality was defined as death occurring during acute hospital stay.

### 2.4. Statistical Analysis

The data was assessed for any missing or outlying data. Frequency distributions were produced for the variables of interest. Continuous variables were presented as means or medians and analyzed using either Student's *t*-test for uniformly distributed variables or Fisher's exact test for nonuniformly distributed variables. Categorical variables were grouped according to the literature standards and expert opinion and were presented as proportions and compared using either a Chi square or Fisher's exact test.

A multivariable logistic regression analysis was performed to determine the variables associated with acute hospital mortality. Variables were chosen a priori based on both previous literature and results of univariable analysis where a *p* value less than 0.1 was used as a cutoff for entry into the model. Hypothesis testing was performed using likelihood ratio test. Statistical analyses were performed using Stata Version 10.1 (StataCorp LP, College Station TX).

## 3. Results

During the study period, 76 patients from three ICUs met our inclusion criteria. All three ICUs were housed in university-affiliated hospitals. The breakdown of biopsies per center was uniform (see Table 1 in Supplementary Materials available online at http://dx.doi.org/10.1155/2016/8715024). Each ICU was a closed/intensivist run ICU with an average of 12 beds per ICU. The median age for the cohort was 63 years (Table 1). The median APACHE II score and PaO_2_/FiO_2_ ratio were 22 and 141, respectively. Twenty patients (26%) were on immunosuppressive therapy prior to ICU admission. This included 3 patients after bone marrow transplant, 3 patients with HIV, 1 patient with a liver transplant, and the 13 remaining patients receiving chemotherapy. In terms of ventilator support, 67% of patients required some form of ventilator assistance on admission to ICU, while all patients except for one were invasively ventilated after biopsy. The median time between biopsy and ICU admission was 2 days (interquartile range: 1–7 days). The majority of patients received an antibiotic (97%) and corticosteroids (70%) while in the ICU prior to biopsy.

The most common histological diagnosis for the whole cohort was diffuse alveolar damage (32%) with no specific etiology attributed. The diagnostic yield of the open lung biopsy was 68% whereby 52 patients had a pathological diagnosis other than diffuse alveolar damage (Table 2). The diagnostic yield of the biopsy did not vary with the time to biopsy. Patients who received a biopsy after five days of admission to ICU had a diagnostic yield of 75% as compared to 65% for patients who had a biopsy before day 5 (*p* = 0.32).

Nine of the 15 patients (60%) diagnosed with an infection had* Pneumocystis jiroveci* pneumonia identified on biopsy. Four of these 9 patients had either a hematological malignancy or HIV and 2 of the 9 patients were on immunosuppressant medications prior to ICU admission. Seven of the 9 patients had a bronchoscopy with bronchoalveolar lavage and one patient had a transbronchial biopsy demonstrating normal lung parenchyma. All of the lavage specimens were examined by a cytopathologist and aside from one sample were negative. The one sample was remarked to have scant PCP but the team did not go on to treat the patient until after the biopsy results.

After diffuse alveolar damage, interstitial lung disease was the most common histological category. Cryptogenic organizing pneumonia was the most common histological diagnosis found in 43% of patients identified with an interstitial lung disease. This was followed by usual interstitial pneumonia, which was found in 35% of patients with interstitial lung disease.

Prior to open lung biopsy, 52 (68.4%) patients had a bronchoalveolar lavage of which 8 patients had growth of an organism. Three patients had findings that were consistent with the results of the open lung biopsy (*Aspergillus*, tuberculosis, and cytomegalovirus). Of the 13 transbronchial biopsies performed prior to open lung biopsy, one patient had an abnormal result demonstrating pneumonitis. The open lung biopsy confirmed a diagnosis of nonspecific interstitial pneumonitis. The decision to proceed with an open lung biopsy was taken by the attending physician and was done to confirm a diagnosis that was felt to be unclear.

**Figure 1:**
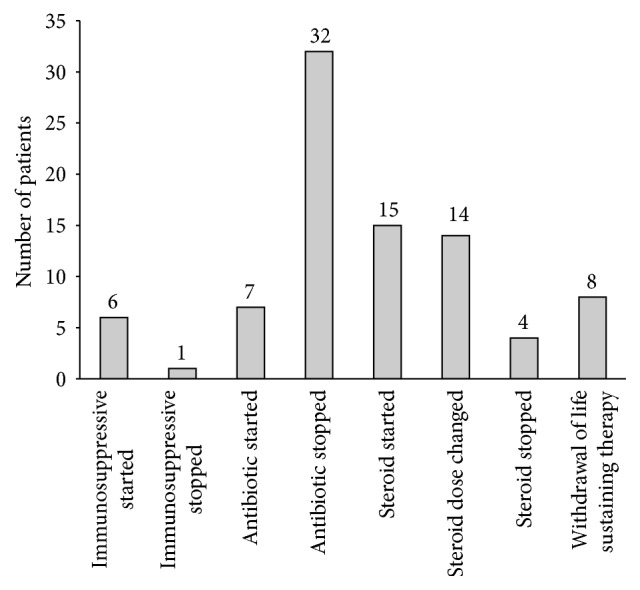
Change of therapy as a direct result of biopsy finding.

The results of the open lung biopsy resulted in a change of therapy for 51 patients (67%) (Figure 1). Fifteen patients (17%) were started on corticosteroid therapy, while six patients (7%) were started on other immunosuppressive medications. The results of open lung biopsy aided in the decision to withdraw life support therapy in eight patients (10%). Thirty-seven (49%) patients had either their antibiotic (32 patients), their corticosteroid therapy (4 patients), or their immunosuppressive therapy (1 patient) stopped due to open lung biopsy results.

Acute hospital mortality was 45%. The median ICU length of stay was 12 days (Table 3). Common causes of death were respiratory failure (22%) and withdrawal of life support therapy (11%). The biopsy results of the eight patients who had withdrawal of life support therapy demonstrated usual interstitial pneumonia (6 patients) and malignancy (2 patients). Complications arising from the biopsy occurred in 30% of patients. Persistent air leaks occurred in 18% of patients while postoperative bleeding occurred in 16%. Two patients had hemorrhagic shock from the biopsy requiring reoperation.

A multivariable analysis of clinical variables associated with acute hospital mortality (Table 4) demonstrated that postbiopsy corticosteroid use was significantly associated with improved survival (OR 0.24, 95% CI 0.06–0.96, *p* = 0.04).

## 4. Discussion

In this study of patients with hypoxic respiratory failure, open lung biopsy resulted in a diagnostic yield of 68% and in a change of management in 67% of patients. We also demonstrated the importance of biopsy results in end-of-life decision-making in that 10% of our patients had their goals of care changed to promote comfort measures. Finally, the use of steroids was the most significant variable associated with survival after open lung biopsy.

Several limitations of this study are worth noting. First, the retrospective nature of the study limits our ability to draw any conclusions with any results being hypothesis generating. Furthermore, given that there was no comparative group of patients with hypoxic respiratory failure who did not receive an open lung biopsy we were unable to demonstrate a mortality difference from the procedure. Such comparisons will require prospective studies to accurately identify patients. In order to better assess the use of the open lung biopsy we used surrogate measures such as change in therapy to gauge the possible benefit from the procedure. As change in therapy in no way guarantees mortality benefit for the patient, it was not possible to fully ascertain the benefit or harm of a biopsy. It is also worth noting that during the time of the study PCR for* Pneumocystis jiroveci* was not available at the study sites and may have led to a decision to go ahead with a biopsy.

Our results are in keeping with those of previous studies in the literature (Tables 5 and 6). We searched the literature for studies that had a similar study question to ours. Specifically, we looked at studies that included critically ill patients with hypoxic respiratory failure and pulmonary infiltrates. Using these criteria we identified 18 studies in the literature of varying sample size from 113 patients to 14 [3, 4]. The mean patient age varied from 37 to 65 years and the majority of studies had 100% of their patients ventilated prior to biopsy, with a few notable exceptions where rates were below 60% [5–7]. Surgical complications ranged in definition and incidence with rates varying from 7% to 56%. For example, Patel et al. reported complications in 39% of patients with the majority being persistent air leaks [8]. Kao et al. reported a complication rate of 20% and resulting mostly from pneumothoraces and transient air leaks [9]. Baumann et al. reported that 33% of patients developed a postoperative pneumothorax with 11% developing a persistent air leak [10]. The wide range of complications across studies could be related in part to differences in defining complications. For example, Lim et al. reported a 56% complication rate but grouped minor and major complications together. In our study we found postoperative complications in 30% of patients, mainly in the forms of bleeding and air leak. A specific diagnosis was found in greater than 50% of patients in all studies except for four studies which had rates in the 40% range [6, 7, 9, 11]. Treatment was altered in more than 50% of patients for 14 of the 18 studies. Data on steroid use and dose change was available for some studies but was largely not available.

Although limited by small number, the multivariable analysis for acute hospital mortality in our study demonstrated that having a steroid responsive disease and receiving steroids was strongly associated with improved outcome. It is worth noting that 70% of patients in our cohort received empiric systemic steroid therapy prior to open lung biopsy. It is conceivable, given that 20% of patients ended up having an infection as the cause of their hypoxic respiratory failure, that the steroids might have had an adverse impact. The role of steroids in pulmonary infections is controversial as it seems to be helpful in some cases, such as* Pneumocystis* pneumonia and community acquired pneumonia [12], and harmful for others like invasive aspergillosis [13]. Given that only one of our patients ended up having aspergillosis it is unlikely that the overall effect to the total cohort was harmful.

Currently, the role of steroids in treating patients with ARDS is unclear with proponents on both sides arguing for or against the use of steroids [1]. The point could be made that empiric treatment of patients with steroids may reveal a steroid responsive disease process and spare the patients the potential complications of an open lung biopsy. It is likely that given our study population of patients with hypoxic respiratory failure and bilateral infiltrates of unknown etiology we studied a higher proportion of steroid responsive diseases than would otherwise have been included in an ARDS study. This may explain the improved outcome observed with steroid use in our study as compared to that seen in the ARDS literature. It is also possible that a policy of liberal empiric steroid therapy may result in more infectious complications, as well as ICU acquired weakness, and lead to poorer overall outcomes. As we did not study a control group of patients with hypoxic respiratory failure and pulmonary infiltrates that did not undergo an open lung biopsy, we cannot determine the present rate of empiric therapy or the merits of such a treatment plan.

Contrary to our results, a study by Papazian et al., which examined 100 patients with ARDS who underwent open lung biopsy [14], found that steroid therapy after biopsy was not associated with improved survival. It should be noted that there was a trend towards improved mortality and the lack of statistical significance may have been due to the small number of patients (*n* = 28) who received steroid therapy. It is also difficult to interpret their results, as the investigators did not know what proportion of patients received steroids prior to open lung biopsy as part of a sepsis protocol. It is conceivable that some patients received steroids unknowingly and may have derived benefit, thereby biasing their result. Another reason for the differences in steroid effect may be due to patient case mix as our study had a higher proportion of immunocompromised patients (4% versus 26%). Differences in patient characteristics and disease process could explain differences in response to steroid therapy.

The timing of biopsy is often debated amongst clinicians with the general feeling that biopsies that are performed at a later date tend to have less productive yield. Although the timing of open lung biopsy is poorly defined, most of the studies showed no effect of timing of biopsy on clinical outcome. In our study the median time from critical care unit admission to biopsy was 2 days with an interquartile range of 1 to 7 days. There was no difference in the diagnostic yield or mortality between patients who had an early or late biopsy.

## 5. Conclusion

Open lung biopsy had a significant diagnostic yield in patients with acute hypoxic respiratory failure with bilateral chest infiltrate of unknown etiology admitted to the ICU. It led to major changes in management and improved decision-making. There was significant morbidity related to the procedure but no procedure related mortality. The receipt of steroids after biopsy was associated with improvement in acute hospital survival. The risk-benefit ratio of open lung biopsy is still unclear, especially given the availability of newer diagnostic tests and possible empirical therapy with steroids. Further research is needed to elucidate the appropriate patient population in which a biopsy is warranted and the surgical approach.

## Supplementary Material

Supplementary material: The table provides information on the number of open lung biopsies performed by centre throughout the years of the study.

## Figures and Tables

**Table 1:** Baseline clinical characteristics of cohort of patients who underwent an open lung biopsy.

	Total cohort (76)
Age (IQR)	63 (56–71)
Male sex, *n* (%)	41 (54.0)
APACHE II (IQR)	22 (18–27)
PaO_2_/FiO_2_ (IQR)	141 (90–174)
Vasopressor use, *n* (%)	6 (7.9)
Mechanical ventilation on 1st day of ICU admission, *n* (%)	51 (67.2)
Invasive	16 (21.1)
Noninvasive	35 (46.1)
Comorbidities, *n* (%)	
Lung disease	27 (35.5)
Hypertension	28 (36.8)
Diabetes	23 (30.3)
Malignancy	20 (26.3)
Solid organ	6 (7.9)
Hematological	14 (18.4)
Chronic renal failure	12 (15.8)
Dialysis dependent	6 (7.9)
Immunosuppressives prior to ICU admission, *n* (%)	20 (26.3)
Medication received in ICU prior to biopsy, *n* (%)	
Steroid	53 (69.7)
Antibiotics	74 (97.4)
Antiviral	12 (15.8)
Antifungal	16 (21.1)
Imaging pattern^µ^, *n* (%)	
Ground glass opacification	65 (85.5)
Nodules	7 (9.2)
Pleural effusion	18 (23.7)
Other	11 (14.5)
Days from admission to open lung biopsy (IQR)	2 (1–7)

*µ*: as seen on computed tomography by a trained pulmonologist.

IQR: interquartile range.

**Table 2:** Pathological results of open lung biopsy specimens.

Pathological diagnosis	Total cohort (76)
Diffuse alveolar damage, *n* (%)	24 (31.6)
Malignancy^*α*^, *n* (%)	5 (6.6)
Interstitial lung disease, *n* (%)	23 (30.3)
Usual interstitial pneumonia	8 (10.5)
Nonspecific interstitial pneumonia	4 (5.3)
Bronchiolitis obliterans organizing pneumonia	10 (13.2)
Hypersensitivity pneumonitis	1 (1.3)
Infectious disease, *n* (%)	15 (19.7)
*Pneumocystis jiroveci*	9 (11.8)
Cytomegalovirus	2 (2.6)
Invasive aspergillosis	1 (1.3)
Miliary tuberculosis	2 (1.3)
Bronchopneumonia	1 (1.3)
Other, *n* (%)	12 (15.8)
Vasculitis	6 (7.9)
Pneumonitis	2 (2.6)
Amyloidosis	1 (1.3)
Drug toxicity	2 (2.6)
Pulmonary edema	1 (1.3)

*α*: malignancy includes hematological malignancy and solid tumor.

**Table 3:** Clinical outcomes of patients who underwent an open lung biopsy.

	Total cohort (76)
Hospital mortality, *n* (%)	34 (44.7)
Sepsis	7 (9.2)
Hemorrhagic shock^*α*^	2 (2.6)
Respiratory failure	17 (22.4)
Withdrawal of active care	8 (10.5)
Complications, *n* (%)	23 (30.3)
Air leak	14 (18.4)
Bleeding	12 (15.8)
ICU length of stay (IQR)	12 (7–25)

*α*: note that these two patients died of hemorrhagic shock at a date remote from the open lung biopsy and for reasons not related to the biopsy.

IQR: interquartile range; ICU: intensive care unit.

**Table 4:** Multivariable analysis of clinical variables associated with acute hospital mortality.

Variables	Odds ratio	95% confidence interval	*p* value
Postbiopsy steroids	0.24	0.06–0.96	0.04
Postbiopsy complications	2.60	0.81–8.36	0.11
Apache II score	1.06	0.97–1.15	0.19
Mode of ventilation on admission			
Oxygen	1.0	—	0.13
Noninvasive ventilation	4.36	0.89–21.28	
Mechanical ventilation	1.99	0.56–7.04	
Timing of biopsy			
Early	1.0	—	0.15
Late	2.25	0.74–6.83	
Sex (female)	1.32	0.44–3.97	0.62

Variables tested in univariable analysis consisted of the following: history of cardiac disease, history of lung disease, history of cancer, history of connective tissue disease, history of endocrine disorder, history of renal failure, use of steroids or vasopressors or antibiotics or immunosuppressants before or after open lung biopsy, timing of biopsy, postbiopsy complications, mode of ventilation prior to biopsy, and APACHE II score. Variables that demonstrated a *p* value < 0.1 were entered into the multivariable model.

**Table 5:** Overview of preoperative characteristics of relevant studies of open lung biopsy in patients with respiratory insufficiency.

Author, year [reference]	Patients *n*	Mean age	Preoperative ventilation (%)	Mean PaO_2_/FiO_2_	APACHE II score	Pre-OLB steroid (%)	Immunosuppression^*α*^ (%)	Preoperative BAL (%)
Warner et al., 1988 [5]	88	55	25	n/a	n/a	53	93	14
Papazian et al., 1998 [15]	36	59	100	118	n/a	n/a	3	100
Flabouris and Myburgh, 1999 [7]	24	49	58	161	n/a	n/a	29	100
Chuang et al., 2003 [6]	17	37	47	192	n/a	n/a	12	68
Patel et al., 2004 [8]	57	53	96	145	n/a	26	30	77
Kao et al., 2006 [9]	41	55	100	116	22	n/a	41	78
Arabi et al., 2007 [4]	14	51	100	153	23	n/a	n/a	100
Baumann et al., 2008 [10]	27	49	100	188	n/a	n/a	67	100
Lim et al., 2007 [16]	36	58	100	119	17	50	28	86
Charbonney et al., 2009 [17]	19	50	100	119	n/a	53	90	95
Kao et al., 2015 [11]	101	57	100	142	23	38	26	85
Hughes and McGuire, 1997 [18]	27	57	100	n/a	n/a	22	25	85
Lachapelle and Morin, 1995 [19]	31	55	80	n/a	n/a	n/a	59	97
Guerin et al., 2015^*β*^ [3]	113	65	100	114	n/a	n/a	n/a	n/a
Papazian et al., 2007 [14]	100	58	100	129	n/a	0	4	100
Bove et al., 1994 [20]	73	50	n/a	n/a	n/a	n/a	32	n/a
Canver and Mentzer Jr., 1994 [21]	27	51	100	n/a	n/a	59	0	70
Soh et al., 2005 [22]	32	51	100	163	19	41	44^*μ*^	n/a
Present study	76	63	47	141	22	70	26	16

n/a: not available.

*α*: immunosuppression definition includes one or more of the following: chemotherapy during the 60 days before lung biopsy, immunosuppressive medication including long term steroid use, organ transplantation, absolute neutrophil count < 1,000/mL, and/or AIDS.

*β*: baseline data only given for 83 (patients with ARDS as per Berlin definition) of the 113 patients.

*μ*: history of malignant disease or having recently received or were undergoing chemotherapy.

**Table 6:** Overview of postoperative characteristics of relevant studies of open lung biopsy in patients with respiratory insufficiency.

Author, year [reference]	Complication^*β*^ (%)	Specific diagnosis (%)	Treatment alteration (%)	Change in management after open lung biopsy			
				Steroids added (%)	Steroid dose increased (%)	Steroids stopped (%)	Total of patients who received steroids after open lung biopsy; [patients on steroids + added − stopped] (%)
Warner et al., 1988 [5]	19	66	70	n/a	n/a	n/a	n/a
Papazian et al., 1998 [15]	19	75	92	17	n/a	3	n/a
Flabouris and Myburgh, 1999 [7]	17	46	75	54	n/a	4	n/a
Chuang et al., 2003 [6]	24	47	65	n/a	n/a	n/a	n/a
Patel et al., 2004 [8]	7	60	60	46	2	3	n/a
Kao et al., 2006 [9]	20	44	73	n/a	n/a	n/a	61
Arabi et al., 2007 [4]	0	100	71	43	n/a	n/a	n/a
Baumann et al., 2008 [10]	7	70	81	26	26	n/a	n/a
Lim et al., 2007 [16]	56	86	64	42	n/a	n/a	n/a
Charbonney et al., 2009 [17]	26	68	89	5	16	16	42
Kao et al., 2015 [11]	14^*∗*^	44	49	16	n/a	n/a	n/a
Hughes and McGuire, 1997 [18]	37^*∗∗*^	74	85	41	15	4	4
Lachapelle and Morin, 1995 [19]	19^*ψ*^	68	59	16	n/a	n/a	n/a
Guerin et al., 2015 [3]	25^∧^	50	n/a	n/a	n/a	n/a	n/a
Papazian et al., 2007 [14]	11^∧∧^	87	78	28	n/a	n/a	n/a
Bove et al., 1994 [20]	12^∧*∗*^	100^∧*∗*∧^	55	45	n/a	4	4
Canver and Mentzer Jr., 1994 [21]	55^*φ*^	100	67	22	27	n/a	n/a
Soh et al., 2005 [22]	41^*μ∗*^	53	44	19^∧*μ*^	n/a	n/a	n/a
Present study	30	67	67	17	n/a	5	5

n/a: not available, data was not made available in the paper.

*β*: complications definition includes persistent air leak more than 7 days or bleeding requiring a blood transfusion.

*∗*: complications related to surgery included postoperative air leak, pneumothorax, subcutaneous emphysema, bleeding, and wound infection.

*∗∗*: persistent air leak through chest tube postoperatively, postoperative pneumothorax, postoperative hemorrhage (>500 mL blood loss in first 24 h), postoperative myocardial infarction, intraoperative desaturation (oxygen saturation, 90% or PaO_2_, 60 mmHg) and persistent air leak postoperatively, intraoperative hypotension (>20% reduction in blood pressure), and postoperative pneumothorax.

*ψ*: prolonged air leak (>4 days) and massive subcutaneous emphysema.

∧: air leaks (leaky chest tubes without pneumothorax, pneumothoraces requiring chest tubes, subcutaneous emphysema without pneumothorax, and bronchopleural fistula after chest tube removal) and bleeding.

∧∧: required blood transfusion during the 48 hr period following OLB, for a hemothorax, mechanical complication beginning during the 48 hr period following OLB pneumothoraces, and moderate air leaks from operative chest tubes for 24 hrs that did not require surgery.

∧*∗*: persistent air leak (longest air leak lasted 14 days), bronchopleural fistula, and patients requiring reintubation with prolonged mechanical ventilation.

∧*∗*∧: includes interstitial pneumonitis, interstitial fibrosis, Pneumocystis carinii, bronchiolitis obliterans, lung carcinoma, metastatic carcinoma, infectious and other pathological diagnosis on lung biopsy.

*φ*: prolonged air leak requiring prolonged chest tube drainage but no surgical therapy.

*μ∗*: persistent air leak, bronchopleural fistula, empyema, and wound infection.

∧*μ*: added or dose changed.
