# Supplementary material for: The Role of Open Lung Biopsy in Critically Ill Patients with Hypoxic Respiratory Failure: A Retrospective Cohort Study
Source: Can Respir J. 2016 May 4;2016:8715024. doi: 10.1155/2016/8715024 (PMC4904511; doi:10.1155/2016/8715024)
Supplement: Supplementary file 1 — Supplementary material: The table provides information on the number of open lung biopsies performed by centre throughout the years of the study. [file 8715024.f1.docx]

Table 1: Biopsy frequency by date and center

| **Year** | **Center 1** | **Center 2** | **Center 3** |
| --- | --- | --- | --- |
| **2000** | 1 | 0 | 4 |
| **2001** | 2 | 0 | 3 |
| **2002** | 0 | 0 | 3 |
| **2003** | 0 | 3 | 0 |
| **2004** | 5 | 2 | 3 |
| **2005** | 1 | 5 | 0 |
| **2006** | 4 | 1 | 2 |
| **2007** | 1 | 3 | 5 |
| **2008** | 2 | 1 | 1 |
| **2009** | 1 | 5 | 2 |
| **2010** | 3 | 3 | 2 |
| **2011** | 0 | 2 | 1 |
| **2012** | 0 | 1 | 0 |
